# Predictive value of hepatic, hematological, and immunological markers and their temporal dynamics in chronic hepatitis B functional cure

**DOI:** 10.1128/spectrum.01780-25

**Published:** 2025-09-08

**Authors:** Jianyong Zeng, Caixia Zheng, Yincheng Zheng, Xiulan Xue

**Affiliations:** 1 The School of Clinical Medicine, Fujian Medical University, Fuzhou, China; 2 Department of Infectious Diseases, The First Affiliated Hospital of Xiamen University, School of Medicine, Xiamen University 12466, Xiamen, China; 3 Xiamen Quality Control Center of Infectious Diseases, Xiamen, China; Children's National Hospital, George Washington University, Washington, DC, USA

**Keywords:** chronic hepatitis, hepatitis B, interferon, predictors, trajectory analysis

## Abstract

Hepatitis B virus (HBV) infection remains a major global health burden. While interferon-alpha (IFNα) therapy demonstrates antiviral and immunomodulatory effects, reliable prognostic markers for sustained response are needed. Transaminases, hematological parameters, and cytokines may serve as potential predictors, but their dynamic changes during IFNα therapy remain poorly characterized. In this prospective real-world study, 181 chronic HBV (CHB) patients received weekly PEG-IFNα-2b for 48 weeks, with serial assessments of virological (HBV DNA, hepatitis B surface antigen [HBsAg]), biochemical (alanine aminotransferase [ALT], aspartate aminotransferase [AST]), hematological (white blood cell [WBC], platelets), and cytokine (interleukin [IL]-4/6, tumor necrosis factor-alpha [TNF-α], IFN-γ) profiles every 4 weeks. The primary outcome was functional cure of CHB. Statistical analyses included (i) generalized linear models (GLMs) evaluating associations between cure status and predictor trends; (ii) Cox regression identifying prognostic factors; (iii) trajectory clustering of biomarker dynamics. Results indicated that sustained declines in ALT (β = −0.025, *P* = 0.029) and AST (β = −0.033, *P* = 0.039) were predictive of functional cure, as confirmed by Cox models (ALT HR = 1.003, *P* = 0.002; AST HR = 1.004, *P* = 0.013). Additionally, the maximum monthly increase in WBC was independently associated with functional cure (β = 0.380, *P* = 0.009). Elevated baseline IL-4 (HR = 1.144, *P* < 0.001) and longitudinal changes in IL-4/IFN-γ also predicted functional cure (*P* < 0.05), although the inclusion of cytokines did not enhance model performance. Trajectory analysis further identified distinct patterns of ALT, AST, and hematological parameter changes linked to functional cure. In conclusion, ALT and AST remain robust predictors of the functional cure of CHB following IFNα therapy, while dynamic WBC and cytokine profiles provide supplementary prognostic value.

**IMPORTANCE:**

Interferon-alpha (IFNα) therapy, known for its antiviral and immunomodulatory effects, lacks reliable predictors of functional treatment response. This study addresses this gap by systematically analyzing dynamic changes in biochemical, hematological, and immunological markers during IFNα therapy. The findings validate alanine aminotransferase (ALT) and aspartate aminotransferase (AST) as robust predictors for functional cure of chronic Hepatitis B virus (CHB), while highlighting the complementary prognostic value of within-visit fluctuations in white blood cell (WBC) counts and cytokine profiles, particularly IL-4 and IFN-γ. Furthermore, the study identifies longitudinal stability in ALT and AST levels, along with specific hematological patterns characterized by initial decline followed by gradual recovery, as predictors of functional cure. By employing generalized linear models, Cox regression, and trajectory clustering, this research provides a comprehensive framework for understanding functional treatment response dynamics of CHB. These findings are significant for clinicians and researchers aiming to refine therapeutic strategies and achieve better patient outcomes in CHB.

## INTRODUCTION

Hepatitis B virus (HBV) remains a significant global health challenge, with an estimated 316 million people living with chronic HBV infection (CHB) in 2019, accounting for 4.1% of the global population (1–3). CHB is a leading cause of liver-related morbidity and mortality, contributing to approximately 0.9 million deaths annually due to complications such as cirrhosis, hepatic decompensation, and hepatocellular carcinoma (HCC) (4, 5). Despite widespread vaccination programs, effective management of existing CHB cases remains a pressing clinical and public health priority (6, 7).

Interferon-alpha (IFNα) was the first drug approved for the treatment of CHB, and it remains a first-line treatment option for CHB to this day (8). IFNα possesses not only antiviral properties but also the ability to modulate the immune response, potentially facilitating the clearance of HBV-infected cells through this immunomodulatory action (9). However, the clinical application of Peg-IFNα monotherapy is limited by its suboptimal efficacy and adverse effects, which compromise treatment adherence and tolerability. Evidence indicates that only approximately 30% of patients attain a sustained virological response with interferon-based regimens. Consequently, combination therapy with PEG-IFNα and nucleos(t)ide analog (NA) has been widely adopted as a primary practical approach for managing CHB (10). Nevertheless, it is reported that long-term use of NA therapy can induce the emergence of antiviral drug resistance. This is because NA therapy can trigger certain resistance mutations (e.g., mutations in the reverse transcriptase region of the HBV genome), which can compromise the efficacy (11). These challenges highlight the importance of PEG-IFNα therapy in long-term treatment regimens, as it offers a mechanism of action distinct from NAs, potentially reducing the risk of resistance. Therefore, the assessment of prognostic prediction after interferon therapy is crucial for improving the long-term health status of patients with CHB.

Traditionally, alanine aminotransferase (ALT) and aspartate aminotransferase (AST) have been used as key biochemical indicators of liver injury and treatment response (12). These enzyme levels demonstrate strong predictive value for treatment outcomes as they reflect the degree of hepatic inflammation and injury. However, these markers alone may provide an incomplete prognostic assessment, as normal ALT levels do not always exclude significant liver fibrosis, particularly in patients with high viral loads (13). Consequently, additional biomarkers are needed to improve prognostic accuracy.

Emerging evidence suggests that cellular and inflammatory markers may enhance outcome prediction in CHB. Hematological indices such as neutrophil-to-lymphocyte ratio and monocyte-to-lymphocyte ratio (MLR) have demonstrated prognostic value in HBV-related liver disease, with elevated levels correlating with poorer outcomes (14). Platelet parameters, which reflect systemic inflammation, have also been associated with disease progression and treatment response (15). Furthermore, cytokines such as interleukin-6 (IL-6), tumor necrosis factor-alpha (TNF-α), and interferon-gamma (IFN-γ) play critical roles in HBV pathogenesis and therapy. Elevated IL-6 and TNF-α levels correlate with HBV suppression, while IFN-γ and interleukin-4 (IL-4) dynamics have been linked to liver fibrosis progression and treatment efficacy (16, 17).

Additionally, dynamic monitoring of post-treatment changes in transaminases, cellular parameters, and cytokines holds significant clinical value in HBV management. First, fluctuations in transaminase levels serve as crucial indicators of hepatic health status. Longitudinal studies demonstrate that sustained variations in serum HBV DNA and aminotransferase levels constitute independent predictors of hepatocellular carcinoma risk, making regular monitoring essential for chronic HBV carriers (18). Second, rapid changes in leukocyte parameters during interferon therapy for chronic hepatitis B may predict superior sustained virological response rates (19). Regarding cytokine profiles, interferon treatment typically induces elevated levels of IFN-γ, TNF-α, IL-4, and IL-12, while significantly reducing regulatory cytokines like IL-10 and TGF-β. These immunomodulatory shifts potentially enhance immune-mediated HBV clearance mechanisms, thereby facilitating hepatitis B surface antigen (HBsAg) seroclearance (19).

This current study aims to comprehensively evaluate the predictive capacity of transaminases, multiple cellular parameters (including hematological indices and platelet metrics), and cytokine profiles—along with their dynamic changes—for determining treatment outcomes in hepatitis B patients receiving IFNα therapy.

## MATERIALS AND METHODS

### Study population

This study enrolled 181 CHB patients from the First Affiliated Hospital of Xiamen University between December 2021 and May 2024. Following enrollment, participants were monitored at 4-week intervals after the initiation of antiviral therapy until the 32-week mark, with a final follow-up assessment conducted at 48 weeks. Participants in this study all provided informed consent, and the study protocol was approved by the Clinical Research Ethics Committee of the First Affiliated Hospital of Xiamen University (no. 2023-176), ensuring compliance with ethical standards in clinical research.

The criteria for inclusion, exclusion, and loss to follow-up applied in the study were detailed in the Supplemental texts.

### Treatment regimen

This study is a real-world prospective study. The study participants in this research were all patients with CHB. Prior to their enrollment in this study, a survey was conducted, and it was reported that all of them had received NA treatments but failed to achieve a functional cure. Considering their strong desire to achieve a functional cure, sequential therapy was adopted, and they were switched to interferon treatment at the beginning of the study. The patients had discontinued NA treatment before the initiation of this study. All patients enrolled in this study followed the treatment with IFNα-2b (Pegbin, Xiamen Amoytop Biotech Co., Ltd.), which was administered subcutaneously at a dosage of 180 µg per injection once weekly for a minimum duration of 48 weeks. Throughout the 48-week treatment period, participants underwent comprehensive clinical tests at 4-week intervals until the 32-week time point, with a concluding follow-up assessment conducted at 48 weeks. During each visit, a series of tests was performed to monitor treatment safety and identify potential adverse events, including liver function tests, complete blood count, and urinalysis. Additionally, serum levels of HBV DNA, HBsAg, ALT, and AST were measured at every assessment to evaluate therapeutic efficacy and disease progression.

### Laboratory tests

In this study, serum HBV DNA levels were quantified using fluorescence quantitative PCR (FQ-PCR). Hepatitis B serological markers, including HBsAg, were measured employing an electrochemiluminescence immunoassay. Biochemical parameters, such as ALT and AST, were analyzed using a colorimetric method with the BECKMANAU5800 (Beckman Coulter). Complete blood cell counts were obtained using the Sysmex XN9000 automatic hematocyte analyzer. These methodologies ensured accurate and comprehensive assessment of virological, serological, and biochemical profiles in the study participants. Inflammatory cytokine levels, namely, IL-4, IL-6, TNF-α, and IFN-γ, were assessed via flow cytometry.

### Definition of outcome

The primary outcome was the achievement of functional cure of hepatitis B, which was defined based on two criteria: (i) a serum HBsAg level of less than 0.05 IU/mL, or (ii) an undetectable serum HBV DNA level, specifically below 20 IU/mL, as determined by FQ-PCR.

### Statistical analysis

#### Linear regression analysis

Firstly, generalized linear models (GLMs) were employed to evaluate the associations between the functional cure status of CHB and the overall average changes in three categories of predictor variables. We first employed GLMs to evaluate associations between the cure status and average changes in three predictor categories: (i) traditional liver function markers (ALT, AST); (ii) cellular parameters (white blood cell [WBC], neutrophil, lymphocyte, monocyte, eosinophil, basophil, red blood cell [RBC], and platelet counts); and (iii) cytokine levels (IL-4, IL-6, TNF-α, IFN-γ). The average change for each variable was calculated as (final follow-up level − baseline level)/number of follow-up periods. These continuous variables served as independent variables in GLMs, with cure status as the binary dependent variable, implemented using R’s “glm” function (v4.4.2).

Subsequently, we analyzed maximum monthly changes, defined as the largest difference between any two consecutive follow-up measurements. For the extended 32–48 week interval, differences were divided by 4 to obtain monthly-equivalent values. All models were adjusted for age and sex as covariates.

#### Cox regression analysis

To identify predictors of HBsAg loss, univariate Cox proportional hazards regression models were employed by using the “coxph” function from the R package “survival” (version 3.7-0). The above-mentioned three categories of variables were included as predictors in the Cox regression models. Additionally, the differences between each follow-up measurement and its corresponding last follow-up measurement (starting from the second follow-up at the fourth week) for these variables were incorporated as independent variables in the Cox models to evaluate the predictive capacity of their changes during the follow-up periods. For the analyses of the original values, sex and age were included as covariates in the models. For the analyses of the difference values, the original values of the variables were further adjusted, along with sex and age.

#### Analysis of change trajectory

In this part, we visualized the trajectories of the aforementioned predictor variables over the follow-up period for each patient after treatment. Subsequently, a clustering analysis was performed on these trajectories to identify patterns of change in the predictor variables. The primary objective of this analysis was to uncover potential causative trends in the predictor variables that may contribute to the achievement of functional cure states.

To characterize these dynamic changes, we employed the dynamic time warping (DTW) algorithm using the R package “dtw” (version 1.23-1), which effectively measures similarity between temporal sequences while accommodating variations in timing and progression rates (20, 21). Following this, the optimal number of clusters was determined through gap statistical analysis (“factoextra” package, version 1.0.7), with a minimum of three clusters specified for each variable. Subsequent hierarchical clustering was performed using Ward’s method (“stats” package, version 4.3.1) to group trajectories into distinct patterns (22, 23). These trajectory classifications were then incorporated as categorical predictors in generalized linear models to evaluate their association with clinical cure outcomes, while controlling for age and gender. Finally, to visually represent the results, characteristic trajectory plots were generated for each classification based on the centroid curves extracted using the DTW algorithm, illustrating the distinctive patterns of each trajectory category.

All statistical tests were two-sided, and *P* values less than 0.05 were considered statistically significant using R software (version 4.4.2).

## RESULTS

### Characteristics of the study population

**Fig 1:**
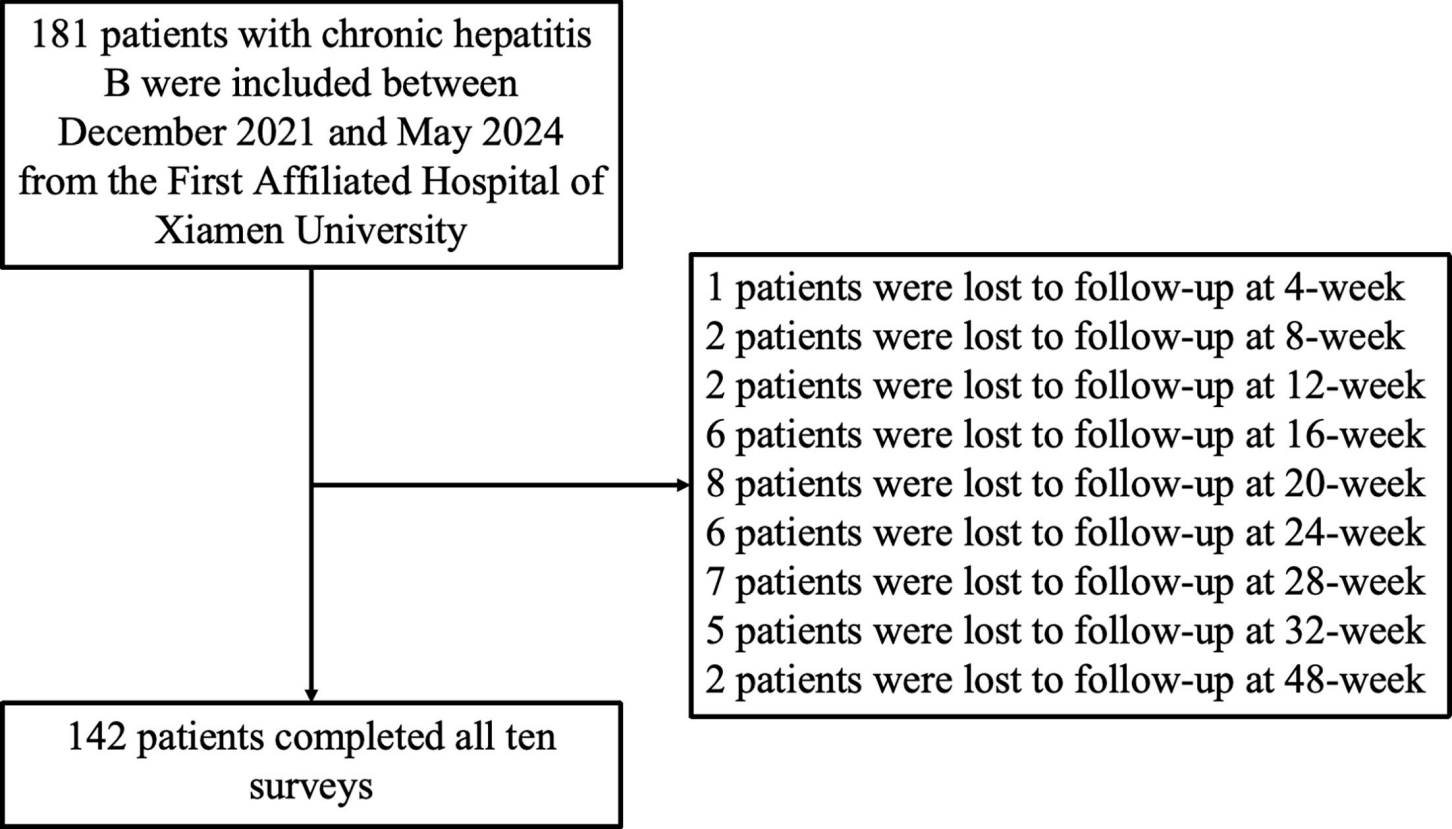
Flow chart of patients in this study.

At baseline, a total of 181 patients with chronic hepatitis B were enrolled in the study. Following nine follow-up sessions—the first eight follow-ups conducted at 4-week intervals and the final session occurring after a 16-week interval—39 participants were lost to follow-up. Consequently, 142 participants completed all follow-up assessments, as illustrated in Fig. 1.

Table 1 summarizes the baseline and 48-week follow-up characteristics of the study population. At baseline, the mean age for the full study population was 38.5, the average HBsAg level was 2.95 log10 IU/mL (SD = 1.37), which significantly decreased to 2.04 log10 IU/mL (SD = 1.69) at follow-up (*P* < 0.01), while HBV DNA levels showed a non-significant reduction from 3.84 log10 IU/mL (SD = 1.93) to 3.11 log10 IU/mL (SD = 1.94) (*P* = 0.09). Among liver function markers, AST levels increased significantly (*P* = 0.05), whereas ALT levels remained stable (*P* = 0.61). In hematological indices, significant reductions were observed in white blood cell count, lymphocyte count, monocyte count, eosinophil count, and platelet count (*P* < 0.01 for all), with other parameters showing less pronounced changes. Among cytokines, IL-6 levels increased significantly (*P* < 0.01), while IL-4, TNF-α, and IFN-γ levels remained largely unchanged. Notably, the withdrawal of some patients due to recovery may have influenced these results, potentially introducing bias in the observed mean values.

**TABLE 1:** Characteristics of the study population^*a*^^,*b*^

	Baseline	Final follow-up	*P* value
N	181	142	
Age, year (SD)	38.54 (8.68)	38.23 (8.90)	0.76
Male (%)	100 (55.25)	79 (55.63)	1.00
HBsAg, log10 IU/mL (SD)	2.95 (1.37)	2.04 (1.69)	<0.01
HBV DNA, log10 IU/mL (SD)	3.84 (1.93)	3.11 (1.94)	0.09
ALT, U/L (SD)	66.55 (92.51)	61.54 (66.36)	0.61
AST, U/L (SD)	45.05 (46.15)	56.53 (44.33)	0.05
WBC count (SD)	5.79 (1.79)	3.24 (1.19)	<0.01
Neutrophil count (SD)	6.06 (34.47)	1.68 (0.80)	0.10
Lymphocyte count (SD)	2.02 (1.62)	1.13 (0.48)	<0.01
Monocyte count (SD)	0.61 (1.10)	0.37 (0.12)	<0.01
Eosinophil count (SD)	0.16 (0.34)	0.05 (0.09)	<0.01
Basophil count (SD)	0.05 (0.12)	0.11 (0.88)	0.58
RBC count (SD)	7.08 (20.22)	4.11 (0.93)	0.05
Platelet count (SD)	230.23 (62.09)	138.18 (40.64)	<0.01
IL-4 (SD)	1.54 (4.40)	0.91 (0.77)	0.14
IL-6 (SD)	3.02 (4.22)	5.18 (5.10)	<0.01
TNF-α (SD)	1.12 (0.87)	1.34 (1.03)	0.13
IFN-γ (SD)	1.79 (1.95)	1.88 (2.67)	0.81

^*a*^ Variables are expressed as mean (standard deviation) or n (%).

^*b*^ HBsAg, hepatitis B surface antigen; HBV DNA, hepatitis B virus DNA level; AST, aspartate aminotransferase; ALT, alanine aminotransferase; WBC, white blood cell; RBC, red blood cell; IL-4, interleukin 4; IL-6, interleukin 6; TNF-α, tumor necrosis factor-α; IFN-γ, interferon-γ.

### Associations between CHB functional cure status and changes in aminotransferase levels, cellular parameters, and cytokines

Firstly, the overall changes in these predictor variables during the whole follow-up periods were included in the GLMs as the independent variables to evaluate their relationships with cure states. From the results of the GLM analysis, it was observed that only the overall changes in ALT (β = −0.025 [95% CI: −0.050 to −0.006], *P* = 0.029) and AST (β = −0.033 [95% CI: −0.068 to −0.006], *P* = 0.039) were significantly associated with the final cure outcome (Table 2).

**TABLE 2:** Associations between the functional cure status of hepatitis B and the overall changes in its predictors^*a*^^,^^*b*^

Predictors	Estimate	95% CI	*P* value
ALT	−0.025	−0.050 to −0.006	**0.029**
AST	−0.033	−0.068 to −0.006	**0.039**
WBC count	0.612	−0.293 to 1.535	0.185
Neutrophil count	0.019	−0.381 to 0.386	0.914
Lymphocyte count	0.263	−1.368 to 1.865	0.708
Monocyte count	0.533	−1.054 to 2.909	0.560
Eosinophil count	−3.485	−14.478 to 4.224	0.452
Basophil count	1.247	−5.100 to 14.538	0.760
RBC count	−0.209	−1.000 to 0.060	0.500
Platelet count	0.017	−0.002 to 0.039	0.092
IL-4	−1.886	−4.498 to 0.106	0.123
IL-6	0.278	−0.061 to 0.818	0.271
TNF-α	−0.536	−2.170 to 0.812	0.434
IFN-γ	0.609	−1.011 to 2.427	0.478

^*a*^ AST, aspartate aminotransferase; ALT, alanine aminotransferase; WBC, white blood cell; RBC, red blood cell; IL-4, interleukin 4; IL-6, interleukin 6; TNF-α, tumor necrosis factor-α; IFN-γ, interferon-γ.

^*b*^ Bold value indicates significant *P* value.

Subsequently, the maximum monthly changes in these predictor variables were incorporated into the GLMs as independent variables. In comparison to the preceding analytical step, a significant association was identified between the maximum change in WBC count and the final cure status of hepatitis B (β = 0.380 [95% CI: 0.106–0.681], *P* = 0.009). In contrast, the associations for ALT and AST were not statistically significant (Table 3).

**TABLE 3:** Associations between the maximum monthly changes in the predictors of hepatitis B and the functional cure status of CHB^*a*^^,^^*b*^

Predictors	Estimate	95% CI	*P* value
ALT	<0.001	−0.004 to 0.004	0.924
AST	−0.002	−0.008 to 0.004	0.562
WBC count	0.380	0.106 to 0.681	**0.009**
Neutrophil count	0.028	−0.055 to 0.126	0.500
Lymphocyte count	−0.072	−0.478 to 0.022	0.432
Monocyte count	0.023	−0.053 to 0.132	0.546
Eosinophil count	−0.218	−1.408 to 0.084	0.640
Basophil count	−0.009	−0.206 to 0.005	0.798
RBC count	−0.025	−0.055 to 0.007	0.576
Platelet count	0.002	−0.003 to 0.006	0.409
IL-4	0.024	−0.155 to 0.186	0.758
IL-6	0.006	−0.008 to 0.025	0.393
TNF-α	0.008	−0.154 to 0.147	0.912
IFN-γ	0.048	−0.016 to 0.161	0.225

^*a*^ AST, aspartate aminotransferase; ALT, alanine aminotransferase; WBC, white blood cell; RBC, red blood cell; IL-4, interleukin 4; IL-6, interleukin 6; TNF-α, tumor necrosis factor-α; IFN-γ, interferon-γ.

^*b*^ Bold value indicates significant *P* value.

### Assessment of the predictive capacity of aminotransferase levels, cellular parameters, and cytokines for functional cure status

In the Cox proportional hazards model analysis, ALT (HR = 1.003 [95% CI: 1.001–1.005], *P* = 0.002), AST (HR = 1.004 [95% CI: 1.001–1.007], *P* = 0.013), and IL-4 (HR = 1.144 [95% CI: 1.084–1.208], *P* < 0.001) demonstrated strong predictive capabilities for the functional cure of CHB. No significant associations were observed with other variables (Table 4).

**TABLE 4:** Results of Cox regression analyses for predictors of hepatitis B functional cure status^*a*^^,^^*b*^

Predictors	HR	95% CI	*P* value
ALT	1.003	1.001–1.005	**0.002**
AST	1.004	1.001–1.007	**0.013**
WBC count	0.992	0.821–1.198	0.931
Neutrophil count	1.007	0.986–1.028	0.535
Lymphocyte count	0.764	0.449–1.302	0.323
Monocyte count	0.991	0.858–1.145	0.903
Eosinophil count	0.705	0.103–4.849	0.723
Basophil count	0.856	0.400–1.834	0.689
RBC count	0.995	0.844–1.174	0.956
Platelet count	1.000	0.996–1.005	0.924
IL-4	1.144	1.084–1.208	**<0.001**
IL-6	0.999	0.987–1.011	0.853
TNF-α	1.051	0.948–1.165	0.348
IFN-γ	0.993	0.910–1.084	0.877

^*a*^ AST, aspartate aminotransferase; ALT, alanine aminotransferase; WBC, white blood cell; RBC, red blood cell; IL-4, interleukin 4; IL-6, interleukin 6; TNF-α, tumor necrosis factor-α; IFN-γ, interferon-γ.

^*b*^ Bold value indicates significant *P* value.

The difference between each follow-up visit (starting with the second follow-up) was then incorporated in the Cox regression models to assess the predictive ability of the changes in these predictors. In this section of the analysis, the differences in ALT and AST levels between follow-up visits demonstrated significant predictive capacity for functional cure status of hepatitis B prior to adjusting for their original values (ALT, HR = 1.005 [95% CI: 1.002–1.008], *P* = 0.002; AST, HR = 1.005 [95% CI: 1.000–1.009], *P* = 0.045). However, this predictive ability became non-significant after adjusting for the original values. On the other hand, the differences in IL-4 and IFN-γ levels between follow-up visits exhibited significant predictive capacity for hepatitis B’s functional cure status, both before (IL-4, HR = 0.866 [95% CI: 0.748–1.000], *P* = 0.049; IFN-γ, HR = 0.958 [95% CI: 0.929–0.988], *P* = 0.007) and after adjusting for their original values (IL-4, HR = 0.853 [95% CI: 0.748–0.971], *P* = 0.017; IFN-γ, HR = 0.957 [95% CI: 0.929–0.985], *P*=0.003) (Table 5).

**TABLE 5:** Results of Cox regression analyses for difference in predictors between each follow-up visit of hepatitis B functional cure status^*a*^^,^^*b*^

Predictors	HR	95% CI	*P* value	HR	95% CI	*P* value
ALT	1.005	1.002–1.008	**0.002**	1.002	0.999–1.006	0.204
AST	1.005	1.000–1.009	**0.045**	1.002	0.997–1.007	0.392
WBC count	1.017	0.806–1.283	0.889	1.123	0.834–1.513	0.444
Neutrophil count	1.011	0.961–1.062	0.680	0.999	0.940–1.063	0.981
Lymphocyte count	0.998	0.917–1.086	0.960	1.035	0.841–1.273	0.746
Monocyte count	1.018	0.947–1.095	0.629	1.279	0.582–2.808	0.540
Eosinophil count	1.008	0.831–1.222	0.935	1.506	0.148–15.365	0.729
Basophil count	1.000	0.993–1.008	0.905	1.145	0.424–3.095	0.790
RBC count	0.924	0.726–1.178	0.527	0.870	0.597–1.269	0.665
Platelet count	0.998	0.995–1.002	0.382	0.999	0.994–1.003	0.478
IL-4	0.866	0.748–1.000	**0.049**	0.853	0.748–0.971	**0.017**
IL-6	0.997	0.991–1.004	0.425	0.997	0.991–1.004	0.377
TNF-α	0.998	0.905–1.101	0.969	0.953	0.872–1.042	0.290
IFN-γ	0.958	0.929–0.988	**0.007**	0.957	0.929–0.985	**0.003**

^*a*^ AST, aspartate aminotransferase; ALT, alanine aminotransferase; WBC, white blood cell; RBC, red blood cell; IL-4, interleukin 4; IL-6, interleukin 6; TNF-α, tumor necrosis factor-α; IFN-γ, interferon-γ.

^*b*^ Bold value indicates significant *P* value.

Additionally, we conducted multivariate Cox regression models incorporating the four significant predictors identified in the previous analysis step to evaluate whether their combined inclusion could enhance the predictive capacity for cure status. The performance of the predictive models was assessed using Harrell’s C-index. The analysis revealed that the simultaneous inclusion of ALT and AST improved the Harrell’s C-index (0.648 vs 0.646 for ALT alone and 0.631 for AST alone). However, the further addition of IL-4 and IFN-γ did not yield additional improvement in the model’s predictive performance (Harrell’s C-index: 0.642 for the model, including all four predictors) (Table S1).

### Associations between hepatitis B functional cure status and trajectories of changes in aminotransferase levels, cellular parameters, and cytokines

The DTW algorithm was initially employed to analyze the trends in predictor changes during the follow-up periods to capture the dynamic characteristics of trajectories of predictors with time. Subsequently, the optimal number of clusters was determined using the gap statistic method. The estimated optimal cluster numbers were as follows: 3 for ALT, 4 for AST, 3 for WBC, 3 for neutrophil count, 3 for lymphocyte count, 3 for monocyte count, 3 for eosinophil count, 3 for basophil count, 3 for RBC count, 3 for platelet count, 3 for IL-4, 3 for IL-6, 3 for TNF-α, and 3 for IFN-γ (Fig. S1).

After clustering, we investigated the association between different trajectory clusters for each predictor and cure status by incorporating cluster categories as independent variables. Significant associations were observed between functional cure status and six distinct trajectory patterns derived from six predictive variables: ALT, AST, WBC count, neutrophil count, monocyte count, and platelet count (Table S2).

**Fig 2:**
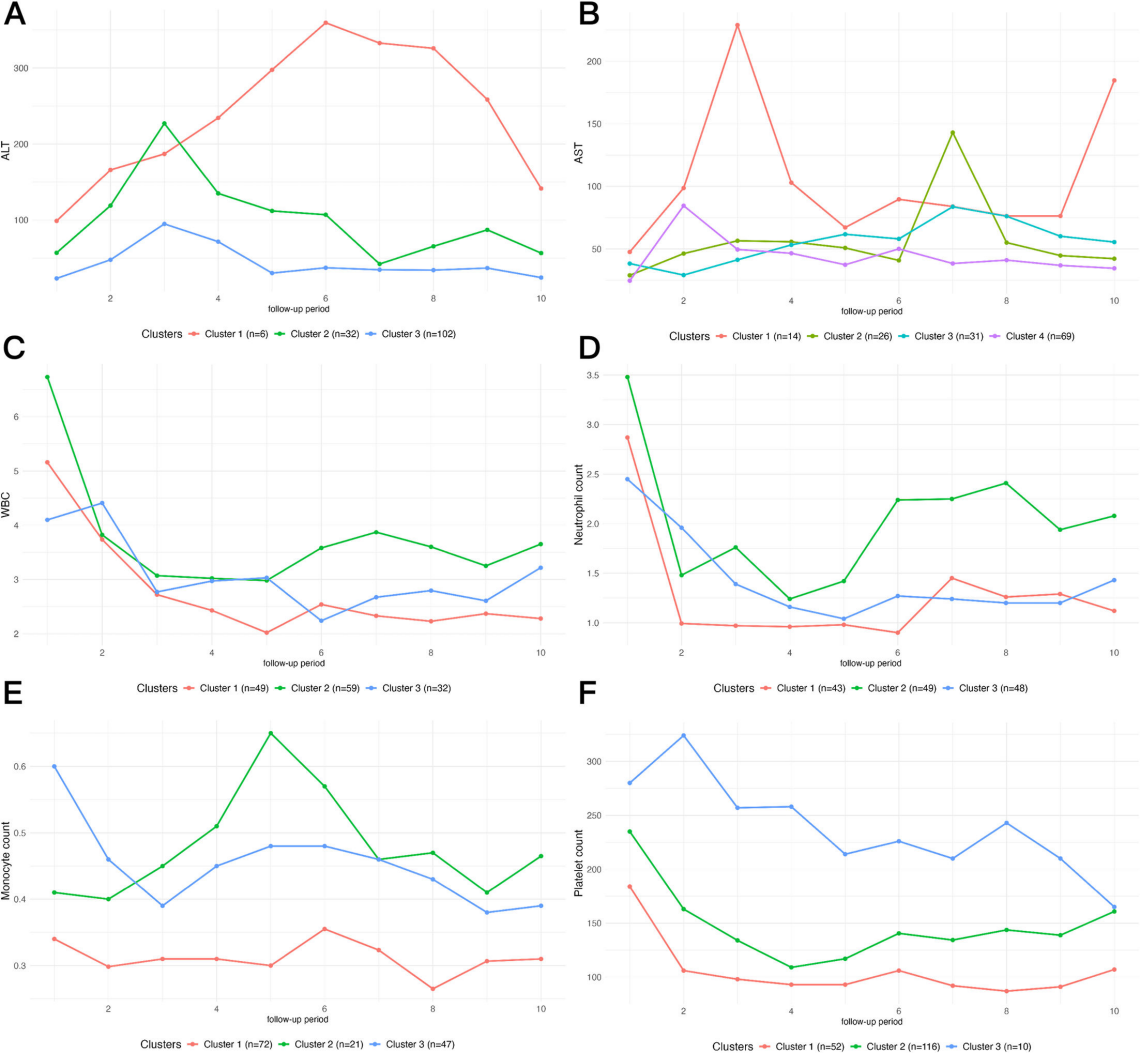
Characteristic curves of clustering categories for the changing trends of several hepatitis B predictors. The curves of clustering categories for the trends of ALT (**A**), AST (**B**), WBC (**C**), neutrophil count (**D**), monocyte count (**E**), and platelet count (**F**).

First, as illustrated in Fig. 2, we identified that trajectory cluster 3 of both ALT (Fig. 2A, *P* = 0.004) and AST (Fig. 2B, *P* = 0.004) showed positive associations with the functional cure of hepatitis B. Compared to other trajectory clusters of these two liver enzymes, these particular clusters exhibited the most stable patterns with minimal fluctuations and consistently lower average levels throughout follow-up. Second, significant positive associations were also found for WBC count trajectory cluster 3 (Fig. 2C, *P* = 0.044), neutrophil count trajectory cluster 3 (Fig. 2D, *P* = 0.026), monocyte count trajectory cluster 3 (Fig. 2E, *P* = 0.007), and platelet count trajectory cluster 2 (Fig. 2F, *P* = 0.050). These significant trajectory clusters shared common characteristics: they all demonstrated initial declines during early follow-up, followed by increases in the later stages, and exhibited more stable patterns with less pronounced fluctuations compared to other trajectory clusters of the same variables (Fig. 2, Fig. S2).

## DISCUSSION

This study systematically evaluated dynamic changes in biochemical, hematological, and immunological markers associated with the functional cure status of hepatitis B using multiple analytical approaches. Key findings demonstrated that (i) generalized linear models identified significant associations between cure outcomes and both overall ALT/AST changes and maximum monthly WBC count fluctuations; (ii) Cox proportional hazards models established ALT, AST, and IL-4 as robust baseline predictors, with within-visit changes in IL-4 and IFN-γ maintaining predictive value after baseline adjustment; and (iii) DTW analysis revealed functional cured patients typically exhibited stable aminotransferase trajectories with minimal fluctuations and characteristic hematological patterns of initial decline followed by gradual recovery.

### Associations between aminotransferase and functional cure status of CHB

ALT and AST are well-established predictors of CHB progression. Elevated levels in CHB patients correlate with histopathological liver changes: ALT associates with hepatic inflammation severity, while AST better reflects fibrosis extent (24). The AST/ALT ratio is a proposed fibrosis marker, though its diagnostic accuracy varies across studies (25, 26). Notably, ALT and AST also participate in immune regulation during HBV infection; ALT fluctuations may mirror immune function changes—a key factor for guiding antiviral therapy (27).

Additionally, elevated ALT and AST levels frequently coincide with metabolic disturbances, such as fatty liver disease and dysregulated glucose/lipid metabolism, which may exacerbate hepatic inflammation and disease progression (28). The predictive value for CHB and HCC risk of dynamic ALT changes has also been demonstrated. Long-term ALT fluctuations independently correlate with HCC, and gradient changes in ALT levels significantly associate with increased risk (18). Virus-induced ALT elevation accompanies rapid HBV DNA rise, whereas host-mediated elevation correlates with gradual DNA decline and improved long-term outcomes (29).

This study not only reaffirms the predictive value of ALT and AST levels in evaluating hepatitis B functional cure but also provides novel evidence linking their dynamic trajectories to CHB prognosis. These findings suggest that monitoring fluctuations in these enzyme levels can enhance the evaluation of disease progression and therapeutic efficacy.

### Associations between hematological parameters and functional cure status of CHB

During the progression of CHB, the counts of various leukocyte subtypes in peripheral blood may hold significant pathophysiological implications. First, neutrophils, as the primary immune defense, exhibit quantitative changes linked to hepatic function and disease burden in HBV infection. Emerging evidence suggests they suppress T-cell responses through arginase-dependent mechanisms, potentially contributing to viral persistence (30). Monocytes are central mediators of inflammation, capable of differentiating into macrophages to modulate tissue repair and immune homeostasis. Among prognostic markers, the MLR demonstrates particular utility in stratifying risks for HBV-related liver complications (31). Moreover, platelet count has been demonstrated to be strongly and inversely correlated with hepatic injury severity, particularly in CHB, where it serves as a non-invasive indicator of liver fibrosis progression. Reduced platelet counts correlate significantly with higher fibrosis-4 index and aspartate aminotransferase-to-platelet ratio index scores, reflecting worsening fibrosis (32, 33). Additionally, thrombocytopenia independently predicts bleeding risk in CHB patients, including upper and lower gastrointestinal hemorrhage (34).

In this study, we identified a strong association between the maximum monthly variation in WBC counts and HBV clearance. Additionally, the dynamic trajectories of WBC, neutrophil, monocyte, and platelet counts demonstrated significant correlations with the functional cure of CHB. It is worth noting that the significant related trends all showed an initial decline in the early post-therapeutic follow-up period, followed by subsequent recovery in later phases. To our knowledge, these findings represent the first reported evidence of such relationships. Although Cox proportional hazards models did not reveal significant predictive value of WBC counts or their subpopulations for HBV clearance, these hematological parameters nevertheless play a crucial role in disease progression.

### Associations between cytokine levels and the functional cure status of CHB

IL-4 and IL-6, as anti-inflammatory cytokines, have demonstrated inhibitory effects on HBV replication. Studies have revealed that IL-4 suppresses the expression of both HBsAg and HBeAg, suggesting its anti-HBV mechanism may involve modulation of viral protein expression (35). IL-6 exhibits multifaceted effects, regulating immune cell functions while contributing to cirrhosis progression and liver injury (36). On the other hand, TNF-α and IFN-γ play critical yet distinct roles in chronic HBV infection progression and clinical outcomes, where elevated TNF-α levels correlate with exacerbated liver injury while increased IFN-γ associates with viral clearance (37). TNF-α mediates liver damage through activation of HBV-specific CD4+ T cells in chronic infection (38). Conversely, IFN-γ directly correlates with viral clearance through HBV-specific CD4+ T cell activation and promotes HBV elimination via immunomodulatory effects on other immune cell populations (37, 39). This functional dichotomy suggests that balanced regulation of these cytokines could represent a promising therapeutic approach for HBV infection.

Our study demonstrates that both IL-4 levels and their longitudinal changes, as well as IFN-γ fluctuations during follow-up, exhibit significant predictive value for the functional cure of CHB. Nevertheless, integrating these variables into existing models incorporating ALT and AST did not further improve predictive performance, suggesting that hepatic and hematological markers may serve as more robust predictors. One explanation is that the immune response triggered by HBV infection involves various cytokines and immune cells, while the alterations in cytokines may not be sufficient to reflect the overall state of the immune system (40). Conversely, ALT and AST, serving as direct markers of liver injury, are capable of more intuitively reflecting the therapeutic efficacy (24). Despite this, the independent prognostic potential of cytokine markers for chronic hepatitis B treatment outcomes merits further investigation.

In this investigation, we employed multiple analytical approaches to comprehensively evaluate the association and predictive capacity of transaminases, various cellular parameters, and cytokines (including their longitudinal changes) with hepatitis B functional cure outcomes. However, several important limitations should be acknowledged. First, as a real-world study, we encountered expected participant attrition due to various clinical and socioeconomic factors. Besides, prior to enrollment, as a large part of the study participants had not undergone their previous treatment at our institution, it was infeasible to acquire and assess their previous clinical indicators. Second, the modest sample size resulted in small subgroup populations for trajectory analysis, potentially affecting result stability. Future validation should be conducted in larger cohorts or through systematic reviews to confirm these preliminary findings. Third, while sufficient for initial analyses, the study duration may have been inadequate to fully assess the long-term predictive value of these biomarkers. In addition, the lack of post-treatment follow-up in this study makes it challenging to reach conclusions about post-treatment management. Finally, this study does not account for the influence of HBV genotypes and intergenotypic recombinations on the severity of CHB and the response to interferon therapy (41). Future research should further investigate their impact based on the findings of this study.

### Conclusions

This study comprehensively evaluated dynamic changes in aminotransferases, hematological parameters, and cytokines as predictors of hepatitis B functional cure. Key findings include (i) ALT and AST levels and their longitudinal changes demonstrated robust prognostic value; (ii) novel identification of dynamic leukocyte counts, IL-4, and IFN-γ changes as significant predictive biomarkers, suggesting immune modulation involvement in HBV resolution; and (iii) characterization of temporal trajectories for multiple parameters (ALT, AST, leukocyte/neutrophil/monocyte/platelet counts) associated with viral clearance. While combining ALT and AST improved prediction, incorporating cytokine dynamics did not enhance model accuracy, suggesting hepatic and hematological markers may be more reliable predictors. These findings highlight the clinical value of multi-parameter longitudinal monitoring for early identification of patients likely to achieve a functional cure.

## Data Availability

The data used in this study is accessible in https://github.com/hongxming/Experimental-data-for-Predictive-Value-for-Chronic-Hepatitis-B-Functional-Cure.

## SUPPLEMENTAL MATERIAL

Supplemental material spectrum.01780-25-s0001.docx Supplemental texts, tables, and figures.

ASM does not own the copyrights to Supplemental Material that may be linked to, or accessed through, an article. The authors have granted ASM a non-exclusive, world-wide license to publish the Supplemental Material files. Please contact the corresponding author directly for reuse.
