# Erratum for Zeng et al., “Predictive value of hepatic, hematological, and immunological markers and their temporal dynamics in chronic hepatitis B functional cure”

**DOI:** 10.1128/spectrum.03374-25

**Published:** 2026-07-10

**Authors:** Jianyong Zeng, Caixia Zheng, Yincheng Zheng, Xiulan Xue

**Affiliations:** 1 Department of Infectious Diseases, The First Affiliated Hospital of Xiamen University, School of Medicine, Xiamen University 12466, Xiamen, China; 2 The School of Clinical Medicine, Fujian Medical University 74551, Fuzhou, China; 3 Xiamen Quality Control Center of Infectious Diseases, Xiamen, China

## ERRATUM

Volume 13, no. 10, e01780-25, 2025, https://doi.org/10.1128/spectrum.01780-25. The affiliations and the affiliation numbers for each author should appear as shown in this erratum.

