# Supplementary material for: Predictive value of hepatic, hematological, and immunological markers and their temporal dynamics in chronic hepatitis B functional cure
Source: Microbiol Spectr. 2025 Sep 8;13(10):e01780-25. doi: 10.1128/spectrum.01780-25 (PMC12502805; doi:10.1128/spectrum.01780-25)
Supplement: Supplemental material — Supplemental texts, tables, and figures. [file spectrum.01780-25-s0001.docx]

**Supplementary Texts**

**Inclusion, exclusion, and dropout criteria in the study**

The inclusion criteria for this study were as follows: (1) patients meeting any of the following conditions: (a) chronic HBV infection, defined as HBsAg positivity for ≥6 months, regardless of HBeAg status or HBV-DNA levels; (b) HBsAg positivity for <6 months with detectable HBV-DNA, accompanied by either (i) persistent or recurrent ALT elevation >2×ULN, (ii) significant necroinflammation on prior liver histology, or (iii) significant fibrosis (>F2) confirmed by histology or non-invasive methods; or (c) HBsAg positivity for <6 months with detectable HBV-DNA plus at least one of the following: (i) family history of HCC, (ii) age >30 years, (iii) evidence of significant liver inflammation (≥G2) or fibrosis (≥F2), or (iv) HBV-related extrahepatic manifestations (e.g., glomerulonephritis, vasculitis); (2) no prior interferon therapy; (3) age 18–65 years; and (4) provision of written informed consent. Exclusion criteria were: (1) coinfection with hepatitis A, C, D, or E viruses or other systemic infections; (2) concurrent liver diseases (autoimmune hepatitis, alcoholic/non-alcoholic steatohepatitis with moderate-to-severe activity, decompensated cirrhosis, or hepatocellular carcinoma); (3) major systemic comorbidities (uncontrolled diabetes, hypertension, severe cardiovascular disease, thyroid disorders, autoimmune diseases, or active psychiatric/neurological conditions); (4) ongoing alcohol abuse, substance dependence, or immunosuppressant use; (5) pregnancy, lactation, or planned pregnancy within 2 years; (6) prior or planned organ transplantation; (7) use of concomitant medications with anti-HBV activity (including herbal therapies); (8) laboratory abnormalities (ALT >10×ULN, total bilirubin >2×ULN, neutrophil count <2×10^9^/L, or platelets <100×10^9^/L); (9) known interferon hypersensitivity; or (10) other investigator-determined contraindications. The dropout criteria were defined as: (1) inadequate adherence to the treatment regimen, indicated by receiving less than 48 weeks of therapy; (2) recovery and discharge of the patient prior to the end of study; (3) occurrence of severe complications or adverse events during the study period; and (4) voluntary withdrawal from participation in the study.

**Supplementary Tables**

**Supplementary Table 1 Harrell's C-indexs for multivariate Cox regression models**

| **Models** | **C-index** | **SD** |
| --- | --- | --- |
| ALT | 0.646 | 0.034 |
| AST | 0.631 | 0.035 |
| IL-4_diff | 0.600 | 0.034 |
| IFN-γ_diff | 0.598 | 0.036 |
| ALT+AST | 0.648 | 0.034 |
| ALT+AST+IL-4_diff | 0.642 | 0.031 |
| ALT+AST+IFN-γ_diff | 0.640 | 0.032 |
| IL-4_diff+IFN-γ_diff | 0.600 | 0.035 |
| ALT+AST+IL-4_diff+IFN-γ_diff | 0.642 | 0.032 |

*AST, Aspartate aminotransferase; ALT, alanine aminotransferase; IL-4_diff, difference in interleukin 4 levels between follow-ups; IL-6_diff, difference in interleukin 6 levels between follow-ups

**Supplementary Table 2 Trajectory analysis results for predictors**

| **Predictors** | **Sample** | **Estimate** | **SE** | ***P* value** |
| --- | --- | --- | --- | --- |
| **ALT** | | | | |
| Cluster 1 | 6 | -15.732 | 1587.151 | 0.992 |
| Cluster 2 | 32 | 0.938 | 0.530 | 0.077 |
| Cluster 3 | 102 | 3.916 | 1.361 | **0.004** |
| **AST** | | | | |
| Cluster 1 | 14 | 0.399 | 0.870 | 0.6463 |
| Cluster 2 | 26 | 0.483 | 0.680 | 0.478 |
| Cluster 3 | 31 | 0.693 | 0.608 | 0.255 |
| Cluster 4 | 69 | 3.866 | 1.344 | **0.004** |
| **WBC** **count** | | | |  |
| Cluster 1 | 49 | 0.543 | 0.636 | 0.393 |
| Cluster 2 | 59 | 0.800 | 0.612 | 0.191 |
| Cluster 3 | 32 | 2.860 | 1.419 | **0.044** |
| **Neutrophil count** | | | |  |
| Cluster 1 | 43 | 0.151 | 0.607 | 0.804 |
| Cluster 2 | 49 | 0.672 | 0.617 | 0.276 |
| Cluster 3 | 48 | 3.205 | 1.438 | **0.026** |
| **Lymphocyte count** | | | |  |
| Cluster 1 | 49 | -0.164 | 0.579 | 0.776 |
| Cluster 2 | 32 | -0.184 | 0.646 | 0.775 |
| Cluster 3 | 59 | -0.229 | 0.285 | 0.420 |
| **Monocyte count** | | | |  |
| Cluster 1 | 72 | -0.258 | 0.587 | 0.660 |
| Cluster 2 | 21 | 0.976 | 0.660 | 0.139 |
| Cluster 3 | 47 | 3.515 | 1.306 | **0.007** |
| **Eosinophil count** | | | | |
| Cluster 1 | 90 | 0.286 | 0.580 | 0.621 |
| Cluster 2 | 9 | 1.257 | 0.997 | 0.207 |
| Cluster 3 | 40 | 0.275 | 0.287 | 0.338 |
| **Basophil count** | | | | |
| Cluster 1 | 42 | 0.318 | 0.713 | 0.655 |
| Cluster 2 | 64 | 0.468 | 0.649 | 0.471 |
| Cluster 3 | 33 | 0.221 | 0.286 | 0.442 |
| **RBC count** | | | | |
| Cluster 1 | 71 | -1.067 | 0.598 | 0.074 |
| Cluster 2 | 3 | 2.187 | 1.357 | 0.107 |
| Cluster 3 | 65 | 0.296 | 0.304 | 0.330 |
| **Platelet count** | | | | |
| Cluster 1 | 52 | 0.604 | 0.667 | 0.364 |
| Cluster 2 | 116 | 1.379 | 0.723 | **0.050** |
| Cluster 3 | 10 | -1.180 | 0.981 | 0.229 |
| **IL-4** | | | | |
| Cluster 1 | 82 | -18.336 | 1455.398 | 0.992 |
| Cluster 2 | 56 | -18.837 | 1455.397 | 0.989 |
| Cluster 3 | 1 | 14.743 | 1455.398 | 0.992 |
| **IL-6** | | | | |
| Cluster 1 | 23 | -0.805 | 0.929 | 0.386 |
| Cluster 2 | 108 | -1.539 | 0.831 | 0.064 |
| Cluster 3 | 8 | -2.031 | 1.488 | 0.172 |
| **TNF‐α** | | | | |
| Cluster 1 | 91 | -2.435 | 1.542 | 0.114 |
| Cluster 2 | 46 | -1.867 | 1.545 | 0.227 |
| Cluster 3 | 2 | -1.853 | 1.861 | 0.320 |
| **IFN‐γ** | | | | |
| Cluster 1 | 131 | -20.407 | 3956.180 | 0.996 |
| Cluster 2 | 6 | -35.687 | 4208.003 | 0.993 |
| Cluster 3 | 2 | 16.646 | 3956.180 | 0.996 |

* AST, Aspartate aminotransferase; ALT, alanine aminotransferase; WBC, white blood cell; RBC, red blood cell; IL-4, interleukin 4; IL-6, interleukin 6; TNF-α, tumor necrosis factor-α; IFN-γ, interferon-γ


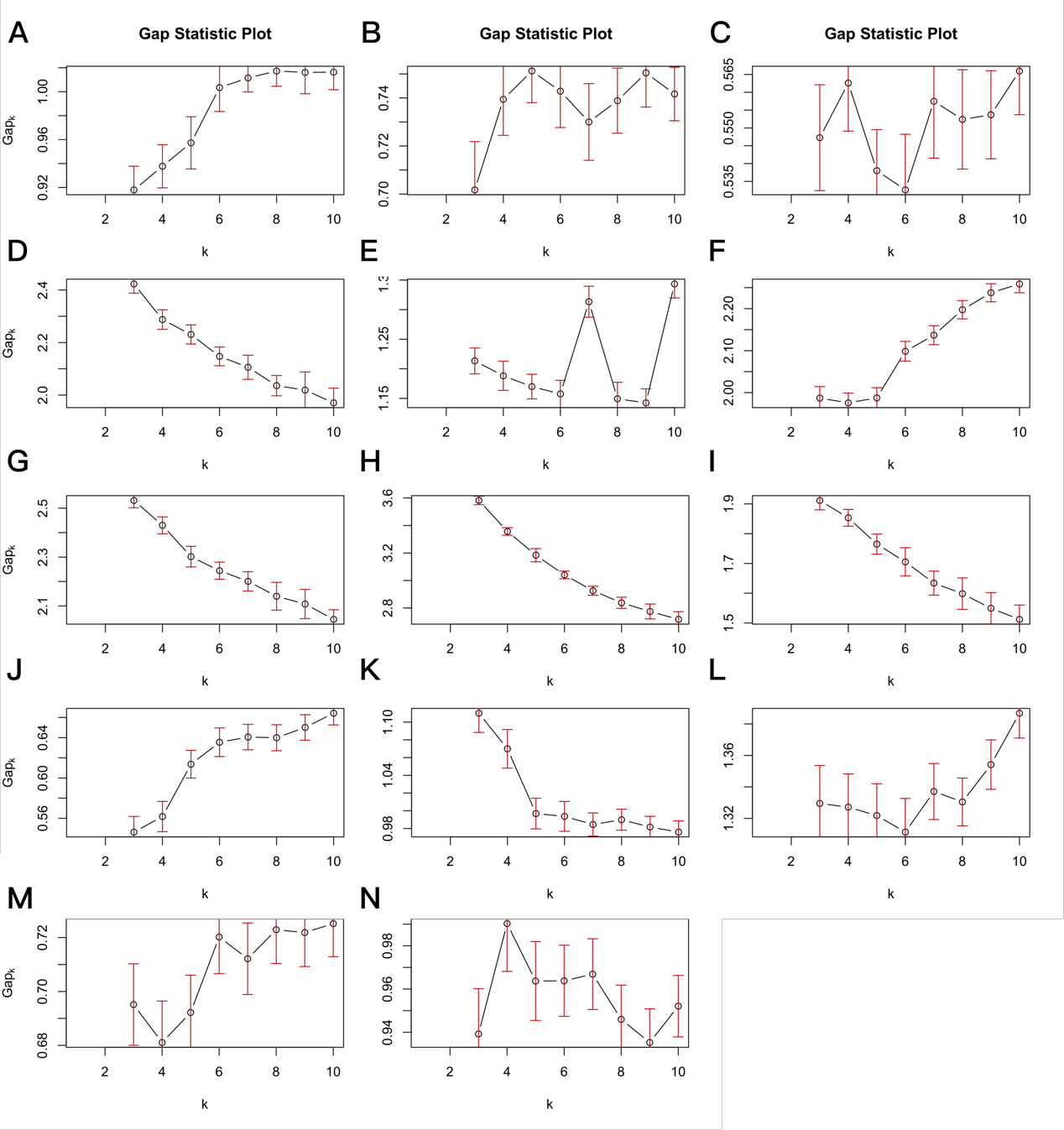


**Supplementary Figure 1 Gap statistic curves for clustering analysis on the trajectory of changes in predictors for hepatitis B cure**

Gap statistic curves for (A) ALT; (B) AST; (C) WBC; (D) Neutrophil count; (E) Lymphocyte count; (F)Monocyte count; (G)Eosinophil count; (H) Basophil count; (I) RBC count; (J) Platelet count; (K) IL-4; (L) IL-6; (M) TNF‐α; (N) IFN‐γ


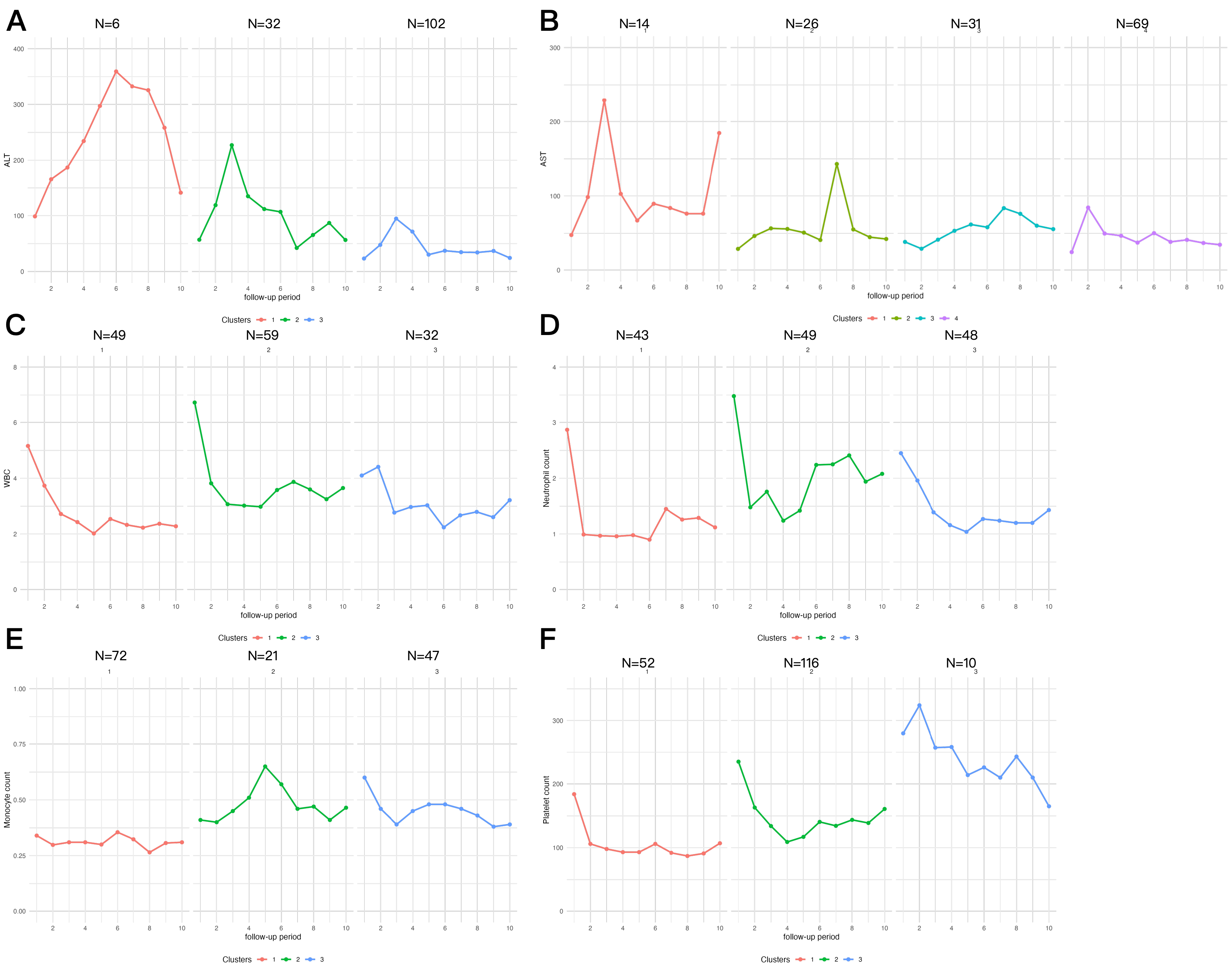


**Supplementary Figure 2 Characteristic curves of clustering categories for the changing trends of several hepatitis B predictors**

* AST, Aspartate aminotransferase; ALT, alanine aminotransferase; WBC, white blood cell
